# A swimming robot actuated by living muscle tissue

**DOI:** 10.1186/1743-0003-1-6

**Published:** 2004-10-28

**Authors:** Hugh Herr, Robert G Dennis

**Affiliations:** 1 Media Laboratory and the Harvard-MIT Division of Health Sciences and Technology, Massachusetts Institute of Technology, Cambridge, MA 02139, USA; 2 Department of Biomedical Engineering, University of North Carolina at Chapel Hill, NC 27599, USA

**Keywords:** Biomechatronics, bionics, cybernetics, hybrid robotics, muscle actuators, skeletal muscle, muscle organ culture, functional electrical stimulation

## Abstract

Biomechatronics is the integration of biological components with artificial devices, in which the biological component confers a significant functional capability to the system, and the artificial component provides specific cellular and tissue interfaces that promote the maintenance and functional adaptation of the biological component. Based upon functional performance, muscle is potentially an excellent mechanical actuator, but the larger challenge of developing muscle-actuated, biomechatronic devices poses many scientific and engineering challenges. As a demonstratory proof of concept, we designed, built, and characterized a swimming robot actuated by two explanted frog semitendinosus muscles and controlled by an embedded microcontroller. Using open loop stimulation protocols, the robot performed basic swimming maneuvers such as starting, stopping, turning (turning radius ~400 mm) and straight-line swimming (max speed >1/3 body lengths/second). A broad spectrum antibiotic/antimycotic ringer solution surrounded the muscle actuators for long term maintenance, ex vivo. The robot swam for a total of 4 hours over a 42 hour lifespan (10% duty cycle) before its velocity degraded below 75% of its maximum. The development of functional biomechatronic prototypes with integrated musculoskeletal tissues is the first critical step toward the long term objective of controllable, adaptive and robust biomechatronic robots and prostheses.

## Background

Many technological barriers exist for the implementation of life-like mobility in robotic and prosthetic systems. Included among these barriers are (1) the availability of high-energy density storage media, (2) the availability of adequate muscle-like actuators, and (3) the availability of biologically inspired sensory technologies. As a possible resolution to these challenges, we consider in this investigation the use of living muscle tissue as a viable actuator for synthetic devices.

Although important research has been conducted to advance a synthetic actuator technology with muscle-like properties, engineering science has not yet produced a motor system that can mimic the contractility, energetics, scalability and plasticity of living muscle tissue [1,2]. Muscle has several important advantages in addition to favorable dynamic characteristics [1-6]. In its function as a motor, muscle acts to provide positive mechanical work at a considerable aerobic transduction efficiency, or 1000 Joules of work per gram of glucose consumed [7]. It is a "smart material", having integrated sensors for the detection of displacement and rate of displacement (muscle spindles) as well as force (Golgi tendon organs). It can repair itself when damaged, and can functionally adapt to an increase in the demands of the environment by undergoing hypertrophic and hyperplastic growth [8] as well as fiber type transformations [9-12]. Muscle has integrated series-elastic components, which are thought to give rise to many of the "life-like" characteristics of animal movement [13], and the fuel that it consumes is a renewable resource, while the waste products produced are environmentally compatible.

In this investigation, we examine the feasibility of using *animal-derived *muscle as an actuator for artificial devices in the millimeter to centimeter size scale. Perhaps researchers in the past did not consider muscle tissue a viable mechanical actuator because of tissue maintenance and control difficulties. The objectives of this study are to identify, and to begin to address, the many technical challenges related to maintaining and controlling explanted muscle tissues in the context of a robotic platform. To this end, we construct a hybrid swimming robot comprising a synthetic elastomeric tail actuated by a single pair of whole muscle explants from frog semitendinosus muscle. We anticipate that basic swimming maneuvers such as straight-line swimming and turning can be performed by alternately modulating electrical signals to each muscle actuator across two electrode pairs, one on each muscle near the neuromotor junction. We further anticipate that a multi-day robotic maintenance or lifespan can be achieved by surrounding the muscle actuators with a specific bath of amphibian ringer's solution comprising antibiotic and antimycotic agents. To test these ideas, we construct two robotic build-ups, each comprising a freshly dissected pair of explanted semitendinosus muscles. For each build-up, pilot data are collected to characterize the robot's swimming mechanics and lifespan.

## Methods

### Muscle Removal and Maintenance

The surgical removal of muscle specimens designated for robotic actuation were performed according to procedures approved by the Committee on Animal Care, Northeastern University (Approval #0402-025-05). Briefly, adult frogs (*Rana pipiens*) were pithed, and both semitendinosus muscles were dissected free and removed with tendons intact. Before removal of the tissues from the animal, the length of each muscle belly was measured at an equilibrium or rest length. The resting length measurement was conducted on the intact muscle specimen with the limb positioned at an anatomically neutral position (see Table I for muscle lengths). After removal from the animal, the muscle, including its intact tendons, was weighed (see Table I for muscle mass). Each tendon was manipulated via tightly secured silk suture (size 5-0). Each muscle was then pinned at its rest length in a 100 mm Petri dish with a previously prepared SYLGARD (Dow Chemical) polydimethylsiloxane (PDMS) substrate.

**Table I:** Muscle actuator parameters and swimming robot performance parameters (mean values, N = 4) at the maximum forward swimming speed for robotic build-ups, B1a and B1b.

Robot	Muscle Mass (g)	Muscle Len. (mm)	Peak Muscle Strain	Muscle Shortening Vel. (mm/s)	Tail-beat Freq. (Hz)	Tail Amp. (mm)	Max. Robot Speed (mm/s)	Wave Speed (mm/s)	Wave Len. (mm)	Slip
B1a	0.31	31	6.5%	25	3.1	16	31	121	39	0.26
B1b	0.34	31	6.5%	25	3.1	16	45	140	45	0.32

Shortly before harvesting the muscles, two fresh liters of amphibian ringer solution were prepared according to a protocol specifically designed for frog organ culture [14,15]. The amphibian ringer comprised: NaCl, 83.89 mM; NaHCO_3_, 28.11 mM; KCL, 1.5 mM; KH_2_PO_4_, 1.2 mM; MgSO_4_, 1.2 mM; CaCl_2 _Dihydrate, 1.3 mM; Glucose, 10 mM; MEM Amino Acid Mixture, 1:50 dilution (GIBCO #1130051); MEM Vitamin Mixture, 1:100 dilution (GIBCO # 1120052); Creatine, 1 mM; DL-Carnitine, 1 mM; Ferric Chloride, 0.9 μM; Human Serum Transferrin, 1.35 μM; Insulin, 1 mU/ml; L-Glutamine, 1:100 dilution; Sigma Chemical #A9909, 1:50 dilution (an antibiotic/antimycotic). A broad-spectrum, antibiotic/antimycotic was added out of necessity for long-term maintenance of the muscles, ex vivo. We observed, for periods greater than 24 hours, septic degradation of the muscle specimens in the absence of the antibiotic/antimycotic agents. After each muscle was placed within a Petri dish, a small volume of ringer solution was used to surround each muscle, the balance being used in the test tank for the swimming robot evaluations. The total amount of time between muscle removal from the animal to finalizing the muscle installation into the robotic swimmer was approximately 1 hour.

### Test Tank Construction and Swimming Robot Design

The test tank was constructed from 6 mm (1/4") thick cast acrylic sheet, welded together with methylene chloride, with silicone fish tank adhesive being applied to form a water-tight seal at each joint. The test tank was 30 cm square and 6 cm deep.

**Figure 1:**
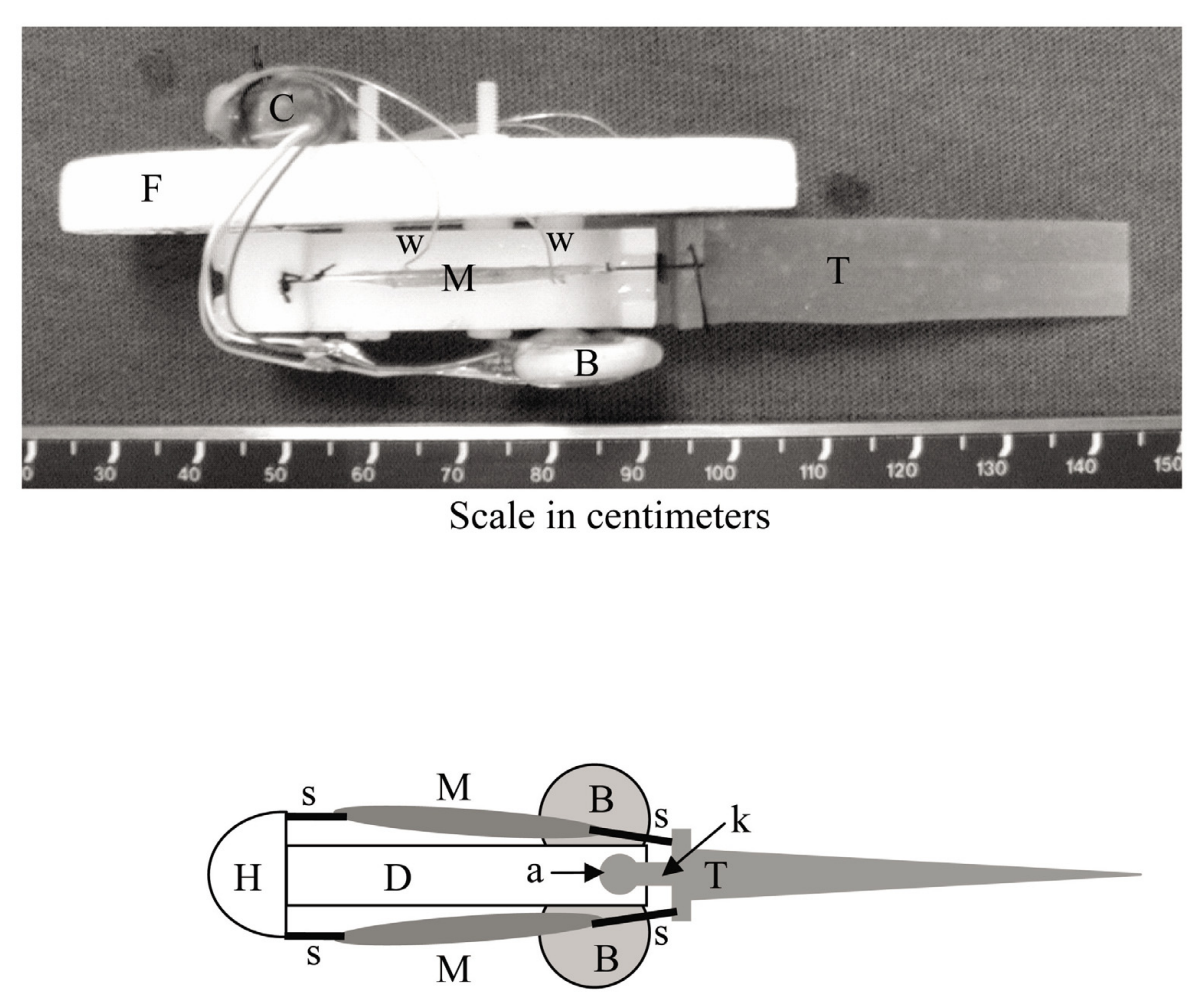
The Biomechatronic Robotic Platform. The top image is a photograph (side view) of the device (robot B1a) shortly after initial testing. The bottom image is a schematic (to scale) with the float and embedded controller removed, showing the main components of the system: semitendinosus muscles (M), suture attachments (s), Styrofoam float (F), electrode wires (w), cast silicone tail assembly (T), rigid Delrin backbone (D), rigid Delrin head piece (H), lithium batteries (B), compliant hinge segment (k), cylindrical tail mounting boss (a), encapsulated microcontroller, infra-red sensor, and stimulator unit (C).

The robotic platform (Figure 1) was specifically designed to accommodate the frog semitendinosus muscles. The actuators were a single pair of whole muscle explants from frog semitendinosus muscle, arranged as antagonists on either side of the robot in an open-frame architecture. This open-frame architecture exposed the explanted tissues to the amphibian ringer solution during robot operations. The robotic platform mass before installation of the muscle actuators was 12.15 g, and the overall length (L) was 12 cm. Of this total length, the fore or anterior 7 cm section comprised a rigid frame machined from acetyl (Delrin) with nylon threaded fasteners, while the aft or posterior 5 cm section comprised a compliant cast silicone tail. A closed-cell Styrofoam float was affixed to the rigid forward section to provide positive buoyancy. The compliant tail had a narrow rectangular section between the mounting flange and the insertion to the rigid Delrin backbone. This compliant segment (Figure 1) served as a hinge for single degree-of-freedom actuation, permitting mediolateral oscillations of the tail. This narrow compliant section also provided a restoring force to return the tail to its neutral position when no muscle force was applied.

The single part silicone RTV (Dow Corning type 734 flowable silicone) tails were cast using a 5-part virgin Teflon mold machined to form a single solid tail assembly with all of the features shown in Figure 1. Casting of one-part silicones was accelerated by the addition of ~1 drop of water-based food coloring per 10 ml of silicone elastomer. This technique allowed tails of different mechanical properties to be readily color-coded during casting, and allowed the elastomer to be fully polymerized and set throughout the entire cross section within 15 minutes of initial mixing. Castings of this sort are not biocompatible for several days due to the emission of acetic acid. If placed in an aqueous environment too quickly with a living tissue, tissue damage would inevitably result. Thicker sections require longer waiting periods, but we found that storage on the shelf for at least one week prior to use was sufficient to achieve biocompatibility with no noticeable effects on the explanted tissues. The cylindrical mounting boss permitted different tail assemblies to be inserted or removed, simply by pressing the boss into a cylindrical receptacle in the Delrin spine. A 0.07 mm diametric interference fit was used. The tail mold allowed different tail lengths and base thicknesses to be cast by simply changing the two Teflon plates that formed the sides of the triangular mold cavity, allowing easy adjustment of the tail compliance. The final tail geometry resulted in sufficient compliance to allow the tail to assume a sigmoidal shape, with a wave traveling caudally when actuated in water at frequencies above ~2 Hz. After design iterations, the spring constant of the compliant tail was 0.42 Newton*cm/radian, and the stiffness remained the same throughout all subsequent experimental sessions.

The onboard electronics were based upon a previously published design for an implantable muscle stimulator [16], and thus the circuit architecture will not be reproduced here. Several minor modifications were made to the circuit hardware. The MAX630 DC-DC converter was not used. The system was powered by two 3 Volt, 48 mAh tabbed lithium batteries (Panasonic # BR1225-1VC) connected in series. The actual operating voltage of the batteries was ~2.8 V [16,17]. The embedded microprocessor (PIC16C54A, SSOP package), was operated from only the first battery in the series, at 2.8 VDC with a 40 kHz crystal oscillator to minimize the power consumption of the device [16,17]. The stimulator output buffer was powered by both lithium batteries in series and was constructed using logic level HEXFETs (International Rectifier # IRF7105) to provide capacitive discharge square pulse stimulation to each actuator at ~5.6 V. The pulse was sufficient to elicit a sub-maximal contraction of each semitendinosus muscle. To minimize the size of the on-board control electronics, a PC board was not used, rather each component was soldered by hand directly to the leads of each IC chip with jumper wires added as necessary.

Stimulation was controlled remotely via a unidirectional infra red (IR) link from a hand-held command module. The on-board fixed stimulation parameters were: amplitude = 5.8 V (alternating bipolar) [16], frequency = 80 Hz, pulse width = 100 μsec. The remote command module allowed for manual control of the onset of stimulation, the train duration (0 to 2550 ms, in 10 ms increments), the dwell time (time between stimulus trains (0 to 2550 ms, in 10 ms increments), and a setting to control either alternating stimulation between the antagonistic actuators for forward motion, or continuous one-sided muscle activation for steering control.

The electrodes were fashioned from medical grade TFE coated 40 AWG stainless steel multi-strand electrode wire (Cooner Wire). The distal ends were stripped to allow the electrode wire to be wrapped around each muscle, as described previously [16]. The finished on-board control modules were encapsulated using electronic grade epoxy, followed by 6 coats of Dow silicone elastomer #734 dispersed with toluene, according to the method described previously [17].

During robotic swimming operations, the fuel sources were a glucose-bearing ringer solution (~2 g/L glucose), and lithium batteries to power the embedded microcontroller and stimulator system. Due to the micro-power electronic design, the estimated battery life for the system was ~21 days (assuming a 10% stimulation duty cycle) [16,17].

The semitendinosus muscle was selected primarily due to its convenient size and tendon anatomy. It is easily dissected with both proximal and distal tendons attached. The proximal tendons of each semitendinosus muscle were sutured to the rigid Delrin head piece, and the distal tendons were sutured to the lateral mounting flange on each side of the tail base using 5-0 braided silk suture (Figure 1). The muscles were mounted symmetrically on opposite sides of the robotic platform to act as antagonists, providing a single degree-of-freedom reversible actuator for the base of the compliant tail. Muscle length was adjusted manually during installation by sliding the sutures through the tail flange to achieve the desired muscle length. Both muscle lengths were adjusted to set each muscle at rest length when the tail was in its neutral position. With no muscle force applied, the restoring torque of the silicone hinge-joint returned the tail to the neutral position, thus both muscles were at their rest length when neither was activated. This important feature is essential for muscle maintenance, as muscles maintained at stretched lengths are known to degenerate more rapidly than muscles held at lengths corresponding to the ascending limb of the length-tension curve [14].

### Robotic Experiments and Performance Characterizations

Two robotic platforms were evaluated in terms of muscle actuator performance, swimming efficiency and locomotory maneuverability. Each robotic platform was designated "B1x", where "x" indicated the build-up, serialized as "a, b, c, ..." for each subsequent pair of explanted frog muscles. Two build-ups were constructed, B1a and B1b, each with a separate pair of freshly explanted frog semitendinosus muscles.

Prior to swimming evaluations, two liters of ringer solution (ringer composition in Methods: *Muscle Removal and Maintenance*) were poured into the test tank, providing a fluid depth of approximately 2.1 cm, enough for the robot to swim without touching the bottom of the tank. The tank temperature was measured but not controlled, and was allowed to stabilize at room temperature, approximately 22°C for the duration of each experiment. The ringer solution was aerated with unfiltered room air using 4 standard porous stone fish tank aerators, one placed at each corner of the tank, and connected to an aquarium aeration pump via silicone tubing. Aeration was discontinued briefly before each test run to minimize turbulence in the test tank. For each robotic build-up, or for each pair of explanted semitendinosus muscles, the test tank ringer solution was not replaced or replenished for the entirety of the robotic experimental session.

Muscle installation was carried out with the robotic platform partially immersed in ringer solution using #5 forceps (Fine Science Tools). After installation was complete, the muscles were allowed to acclimate for a period of approximately 5 minutes before stimulation. The robot was manually placed to allow forward motion through the bath, and muscle stimulus parameters, specifically stimulus train duration and dwell time, were varied manually until the maximum swimming velocity was achieved. To increase swimming speed, dwell period was decreased and train duration was increased until further decreases in dwell time or further increases in train duration did not result in additional increases in forward swimming speed. During experimentation, swimming speed was determined by measuring the amount of time required for the robot to swim across a known, fixed distance. Once the maximum swimming speed was achieved, the ventral view of the swimming robot was filmed (Sony Model #DCR-TRV820; 30 frames/sec), and the film was then digitized to determine tail-beat frequency, tail amplitude, and the wave speed and wave length of the propulsive body wave. In addition to forward straight-line swimming, muscle stimulus parameters were varied to investigate turning maneuvers. At a maximum forward swimming speed, the robot's open loop, alternating stimulation pattern between the antagonistic actuators was switched to a continuous one-sided muscle activation for steering control, causing the robot to turn in the direction of the single stimulated muscle (a medial turn resulting from one-sided medial muscle stimulation). Here again, the ventral view of the swimming robot was filmed, and the film was then digitized to determine the maximum turning radius.

For each tail-beat period, at least 10 video frames were captured, separated in time by 33 ms, depending on the swimming speed of the robot. A customized software program was used to digitize 10 points on each side of the outline of the ventral silhouette of the robot, for a total of 20 points for each image. A series of cubic spline functions were used to draw the best-fit line along these points [18,19], and a midline was constructed. Tail-beat frequency was measured by tracking a digitized point on the tail tip from the ventral view over the course of one tail-beat cycle and dividing by the elapsed time. Tail amplitude was determined by measuring the tip-to-tip linear distance at the two extremes of tail excursion and then dividing by two. As described by [20], mean propulsive wavelength was measured directly from the reconstructed midlines as the distance between two successive peaks present on the robot's body. Propulsive wave speed was calculated by dividing the distance between the anterior most point of the body exhibiting undulation and the tail tip by the time required for the crest of the wave to pass through these points.

To estimate the overall mechanical swimming efficiency of each robotic build-up, we calculated the robot's slip value, a dimensionless velocity [21]. A high slip value indicates a larger contribution to rearward, thrust-producing forces than lateral forces. Slip was calculated by dividing the robot's steady state swimming velocity by its propulsive wave speed.

To estimate muscle actuator performance at the maximum swimming speed, muscle strain and shortening velocity were estimated using the tail-beat frequency and amplitude measurements taken from the digitized films. After the swimming experiments were finalized, the change in linear distance between the robot's muscle attachment points was measured when the robot's tail was re-positioned from a neutral, straight position to the tail amplitude posture measured during straight-line swimming. As an estimate of peak muscle shortening strain, this linear-distance change was then divided by the muscle's resting length, or the muscle belly length when the tail was held straight (resting length measurement protocol defined in Methods: *Muscle Removal and Maintenance*). Still further, to estimate muscle-shortening velocity at the maximum swimming speed, the measured linear-distance change between muscle attachment points was divided by the time required for the tail to re-position from a neutral, straight position to the tail amplitude posture measured during straight-line swimming. This time period was measured from the digitized films and was equal to approximately one quarter of a tail-beat period.

For the turning maneuvers, the turning radius was estimated from the ventral video images by tracking the spatial trajectory of a point midway between the tail tip and the nose of the robot, a distance 6 cm from the tail tip along the midline of the robot when the tail assumed a neutral, straight orientation. The turning radius was the radius of a circle with an arc curvature equivalent to the midpoint trajectory curvature.

### Semitendinosus Contractile Experiment: Maximum Shortening Velocity

To estimate the contractile efficiency of the robotic muscle actuators at the maximum swimming velocity, a separate experiment was conducted to determine the maximum shortening velocity of freshly dissected semitendinosus muscles of comparable size and rest length to that of the muscles employed in robotic build-ups, B1a and B1b. Six freshly dissected semitendinosus muscles were placed in a muscle characterization apparatus (Aurora Model 305B) and isotonic contraction experiments [22] were conducted to measure the muscles' maximum shortening velocity. The contractile experiment was conducted at the same temperature as the robotic experiments, or 22°C.

## Results

### Robotic Performance Characterizations

For the B1a and B1b robotic swimmers, the locomotory performance parameters at maximum swimming velocity are summarized in Table I. Table I also includes the muscle actuator mass and rest length for each robotic build-up. For both robot B1a and B1b, the total muscle mass did not exceed 6% of the total mass of the robot (B1a = 4.8%; B1b = 5.3%). Even with such a low relative actuator mass, swimming robots B1a and B1b achieved top speeds greater than 1/4 and 1/3 body lengths per second, respectively (here the robot's total length, 12 cm, was used as the normalization factor). For both robotic swimmers, forward swimming speed was readily controllable simply by decreasing the dwell period or by increasing the train duration. The maximum steady state, forward swimming speed was achieved with alternating actuator contractions of 110 ms train duration, with 40 ms dwell periods between each stimulus train, resulting in 3.1 tail-beats per second. Further increases in the stimulus train duration or further decreases in the stimulus dwell time did not result in additional increases in forward swimming speed.

Each robotic build-up was capable of the following controlled maneuvers: forward accelerations, decelerations, steady state gliding, and turning to the right or left. The robot was capable of surface swimming only, so all maneuvers were restricted to 2-dimensions. Turning was accomplished after forward momentum had been established by continuously activating only one actuator. The minimum gliding turn radius was 400 mm as estimated from the digitized video images of the robot's midpoint trajectory.

After swimming the full length of the test tank, the robot was manually repositioned to the opposite end of the tank where it began, once again, to swim across the tank width. Typically, a period of swimming activity (~3 min) was followed by a period of swimming inactivity (~30 min). Due to muscle fatigue, periods of inactivity were required to restore the robot's peak swimming velocity to at least 75% of its maximum value measured during the first session of robotic swimming (first 10 minutes of the robot's lifespan). Robot B1a swam for a sum total of 45 minutes over a 7.5 hour lifespan (10% duty cycle), after which its swimming velocity degraded below 75% of its maximum value even after a 30 minute period of swimming inactivity. In distinction, robot B1b swam for a much longer period – a sum total of 4 hours over a 42 hour lifespan (10% duty cycle) before its velocity degraded below 75% of its maximum value following a 30 minute period of swimming inactivity.

To compare the overall swimming efficiency of each robotic build-up, we calculated the propeller efficiency using the measure of slip (swimming velocity/ propulsive wave speed) (Table I). In a steady-state condition, at the maximum forward swimming speed, slip values for robotic build-ups, B1a and B1b, were 0.26 and 0.32, respectively. By comparison, slip values generally increase with swimming speed in fish, ranging from 0.2 to 0.7 in most fish [19,20]. The mechanical swimming efficiency of robots B1a and B1b, as determined by their respective slip values, were within the biological efficiency range.

### Maximum Shortening Velocity and the V/V_max _Ratio at Maximum Swimming Speed

In a separate experiment from the robotic investigations, six freshly dissected semitendinosus muscles (mass = 0.34 ± 0.04 g; rest length = 30 ± 1 mm; Mean ± S.E., N = 6 muscles) produced a maximum shortening velocity, V_max_, of 78 ± 3 mm s^-1 ^(Mean ± S.E., N = 6 muscles) in isotonic contractions. At the maximum swimming speed, the muscle actuators within robots B1a and B1b experienced a shortening velocity of 25 mm s^-1 ^(Table I), giving a V/V_max _ratio of 0.32, an intermediate contraction velocity where muscle typically produces peak power and efficiency [7].

## Discussion

Although a great deal of research has been conducted to advance a synthetic actuator technology with muscle-like properties, engineering science has not yet produced a motor system that can mimic the contractility, energetics, scalability and plasticity of living muscle tissue [1,2]. In this investigation, we examine the feasibility of using *animal-derived *muscle as an actuator for artificial devices. We construct a simple robotic platform powered by explanted living amphibian muscle and controlled by an embedded microcontroller via an infra red data link. Using an open loop control and a simple interface design, we present preliminary data that suggests that living muscle might one day be employed as a practical, controllable actuator. Hybrid robot B1b remained active for up to 42 hours, and during that time, performed basic swimming maneuvers such as starting, stopping, turning and straight-line swimming at speeds exceeding 1/3 body lengths per second. The muscle-actuated swimming robot also offered a reasonable swimming efficiency, as indicated by a slip value of 0.32 (see Table I).

### Muscle Fiber Type and Control

The frog semitendinosus muscles employed in the robot were comprised predominantly of fast-twitch muscle fibers, and therefore provided higher mechanical power, at the expense of being considerably more fatigable, than would have been achievable using a slow-twitch muscle of comparable size. Ideally, a biomechatronic swimming robot would incorporate several muscle fiber types to permit both explosive as well as low-power locomotion and maneuvering. For the robotic platform of this investigation, it is important to note that the stimulation was non-physiologic in many ways. Each muscle was stimulated in bulk, with all fibers being subjected to approximately the same electric field. In living muscle in vivo, individual motor axons innervate one or more muscle fibers, establishing the fundamental neuromotor functional unit: a *motor unit*. In a sophisticated biomechatronic system, a motor-unit level of control would be desirable (fast vs. slow), both for controllability and for tissue phenotype maintenance.

### Tissue Failure Modes

In this study, the performance of the muscle actuators eventually degraded to the point where they were no longer effective mechanical actuators. Several factors contributed to the observed tissue degradation. To begin with, explanted muscle generally has a very finite functional life expectancy [14,15], usually less than one day. Excluding such transient failure modes as metabolic muscle fatigue, the major failure modes of muscle *in vitro *generally fall into one of the following categories: (1) core necrosis due to lack of oxygenation/capillary perfusion and large diffusion distances, (2) sepsis, (3) exogenous toxicity, (4) electro-chemical damage resulting from excessive electrical stimulation, (5) accumulated contraction-induced injury, (6) sarcomeres heterogeneity leading to loss of thick and thin filament overlap in regions of muscle fibers (exacerbated by prolonged periods at or above the optimal length for force generation), and (7) direct mechanical damage to the muscle from external sources, such as the robot frame, attachment hardware, or electrodes.

For the tissue-actuated device of this investigation, several design considerations were made to minimize many of these failure modes. The bath was aerated to assist oxygen delivery to the tissues, although this strategy would only be helpful to the outer shell of muscle fibers no greater than ~200 μm from the surface. In addition, the level of muscle cell depolarization was kept to a minimum in order to limit electro-chemical damage [16]. Still further, the muscle actuators were attached to the robot frame at rest length in order to minimize the risk of excessive muscle strains and sarcomere heterogeneity. Clearly, when looking to the future, other failure modes must be considered when very long periods of *ex vivo *tissue maintenance are necessary. These include loss of muscle excitability and mass, phenotypic drift, and de-differentiation of the muscle from desired adult muscle phenotypes.

### Muscle Actuator Source: Engineered Muscle versus Explanted Tissue

Even though organogenic mechanisms are poorly understood, it is nonetheless possible to engineer functional muscle organs from individual cells in culture [23-26], but currently these tissue constructs have several practical limitations that limit their usefulness as living actuators. Among these limitations are: (1) low contractility, similar to that during early stages of muscle development, (2) low excitability, thus requiring large amounts of electrical energy to adequately stimulate the tissue to contract, (3) the lack of perfusion, which limits the tissue cross section to a maximum radius of approximately 200 μm, and (4) the lack of suitable tissue interfaces, both neural and mechanical. Given such technological limitations, we chose in this study to employ explanted muscle tissues for robotic actuation. However, once these technical hurdles are overcome, engineered muscle actuators might offer important advantages to the construction of biomechatronic robots.

### Future Work

The results of this investigation, although preliminary, suggest that some degree of ex vivo robustness and longevity is possible for natural muscle actuators if adequate chemical and electromechanical interventions are supplied from a host robotic environment. Clearly, an important area of future research will be to establish processes by which optimal intervention strategies are defined for a given hybrid-machine task objective. Another important area of research will be tissue control. It has been established that natural muscle changes in size and strength depending on environmental work-load, and when supplied with appropriate signals, changes frequency characteristic or fiber type [9-11]. Hence, an important area of future work will be to put forth strategies by which muscle tissue plasticity can be monitored and controlled. Finally, strategies must also be devised to control the force and power output of muscle, in the context of robotic systems, through the modulation of electrical pulses to the muscle cell. To achieve the long-term objective of functional, muscle-actuated robotic and prosthetic devices, we feel controlling machine movements through electrical stimulation, harnessing muscle tissue plasticity, and maintaining ex vivo contractility are critical areas for future research.

## Conclusion

In this paper, we ask whether muscle tissue explants can be employed as mechanical actuators for robots in the millimeter to centimeter size scale. Using a very simple control and interface design, we present preliminary data that suggests that living muscle might one day be employed as a practical, controllable actuator. The robot of this investigation remained active for up to 42 hours, and during that time, performed basic swimming maneuvers such as starting, stopping, turning and straight-line swimming at speeds exceeding 1/3 body lengths per second. It is our hope that this work will lead to further studies of tissue actuated robots and prostheses that will result in an even wider range of biomechatronic machine capabilities.
